# Quality assurance in human papillomavirus testing for primary cervical screening

**DOI:** 10.1136/ijgc-2022-004197

**Published:** 2023-03-13

**Authors:** Kate Cuschieri, María Dolores Fellner, Laila Sara Arroyo Mühr, Elizaveta Padalko, Rita Mariel Correa, Joakim Dillner, Murat Gultekin, Maria Alejandra Picconi

**Affiliations:** 1 Scottish HPV Reference Laboratory, Royal Infirmary of Edinburgh, Edinburgh, UK; 2 Argentinian HPV Reference Laboratory, National Institute of Infectious Diseases-ANLIS "Dr Malbran", Buenos Aires, Argentina; 3 International HPV Reference Center, Medical Diagnostics Karolinska, Karolinska Universitetssjukhuset, Stockholm, Sweden; 4 Clinical Science, Intervention and Technology, Karolinska Institutet, Stockholm, Sweden; 5 Belgian HPV Reference Laboratory, University Hospital Ghent, Ghent, Belgium; 6 Turkish HPV Reference Laboratory, Hacettepe University Faculty of Medicine, Ankara, Turkey

**Keywords:** Cervical Cancer, Cervix Uteri

## Abstract

The recommendation for cervical screening is that it should be based on human papillomavirus (HPV) molecular testing. For all screening programs, attention to quality assurance is required to fully realize the benefits. Internationally recognized quality assurance recommendations for HPV-based screening are needed that are ideally applicable for a variety of settings, including in low- and middle-income countries. We summarize the main points of quality assurance for HPV screening, with a focus on the selection, implementation, and use of an HPV screening test, quality assurance systems (including internal quality control and external quality assessment), and staff competence. While we recognize that it might not be possible to fulfill all points in all settings, an awareness of the issues is essential.

## BACKGROUND

Understanding that persistent infection with human papillomavirus (HPV) is a necessary factor for the development of most cervical cancers has informed key developments for prevention. The World Health Organization's (WHO) strategy to eliminate cervical cancer as a public health problem, considers that screening with a high-performance test is one of the three key pillars to support elimination—along with HPV vaccination and treatment—and should benefit all women.^1 2^

Evidence shows that screening using assays to detect HPV nucleic acids is more effective to reduce the incidence of, and mortality from cervical cancer than screening with cytology.^3–5^ A growing array of HPV-based screening tests are available, and a number of countries have now introduced HPV-based cervical screening. In the clinical laboratory, the concepts of quality assurance were first introduced in hematology and clinical chemistry in the late 1940s/early 1950s in an effort to reduce the number of diagnostic errors in the handling and testing of patient specimens, which ultimately could have an impact on patient care. These general principles formed the quality assurance foundation commonly used in the clinical virology laboratory. Additionally, they continue to evolve to meet present regulatory demands and assure the safety and effectiveness of testing services.^6^ Like any other laboratory assay, the delivery of HPV testing has to be monitored to identify areas for improvement and to avoid suboptimal, potentially harmful practice. Robust operations and accurate analyses in the laboratory support optimal care.^7^

This said, we should remember that the interpretation and application of quality assurance criteria is somewhat heterogeneous worldwide. Additioanlly, the number of tests and indeed laboratory services required to fulfill WHO 2030^1^ goals is significant. Some testing may be performed in clinical or pathology laboratories with a mixed experience and awareness of quality assurance systems. Furthermore, countries may choose to establish new centralized screening laboratories or launch more point-of-care/near patient testing systems. Availability of resources may differ greatly between different settings. In some low- and middle-income countries, implementation of quality assurance may be beset with difficulties, such as the availability of economic and material resources, decision-makers' awareness, availability of trainers, and finally the perception of its importance among other competing factors.

The Global HPV Laboratory Network (HPV LabNet) was created by WHO in 2006 to support the worldwide development and implementation of HPV vaccines through improved laboratory standardization and quality assurance of HPV genotyping for research, evaluation of HPV vaccines, and HPV surveillance.^8 9^ However, HPV-based cervical screening is today an important part of the cervical cancer elimination strategy and clearly quality assurance of laboratory processes of HPV testing is increasingly relevant for cervical cancer control.

The current HPV LabNet comprises national HPV reference laboratories appointed by their respective countries and led by the International HPV Reference Center.^10^ The tasks of national HPV reference laboratories were specified by WHO in 2005 and include actively working to support laboratories that perform HPV testing for screening, with particular consideration for quality assurance.

**Figure 1:**
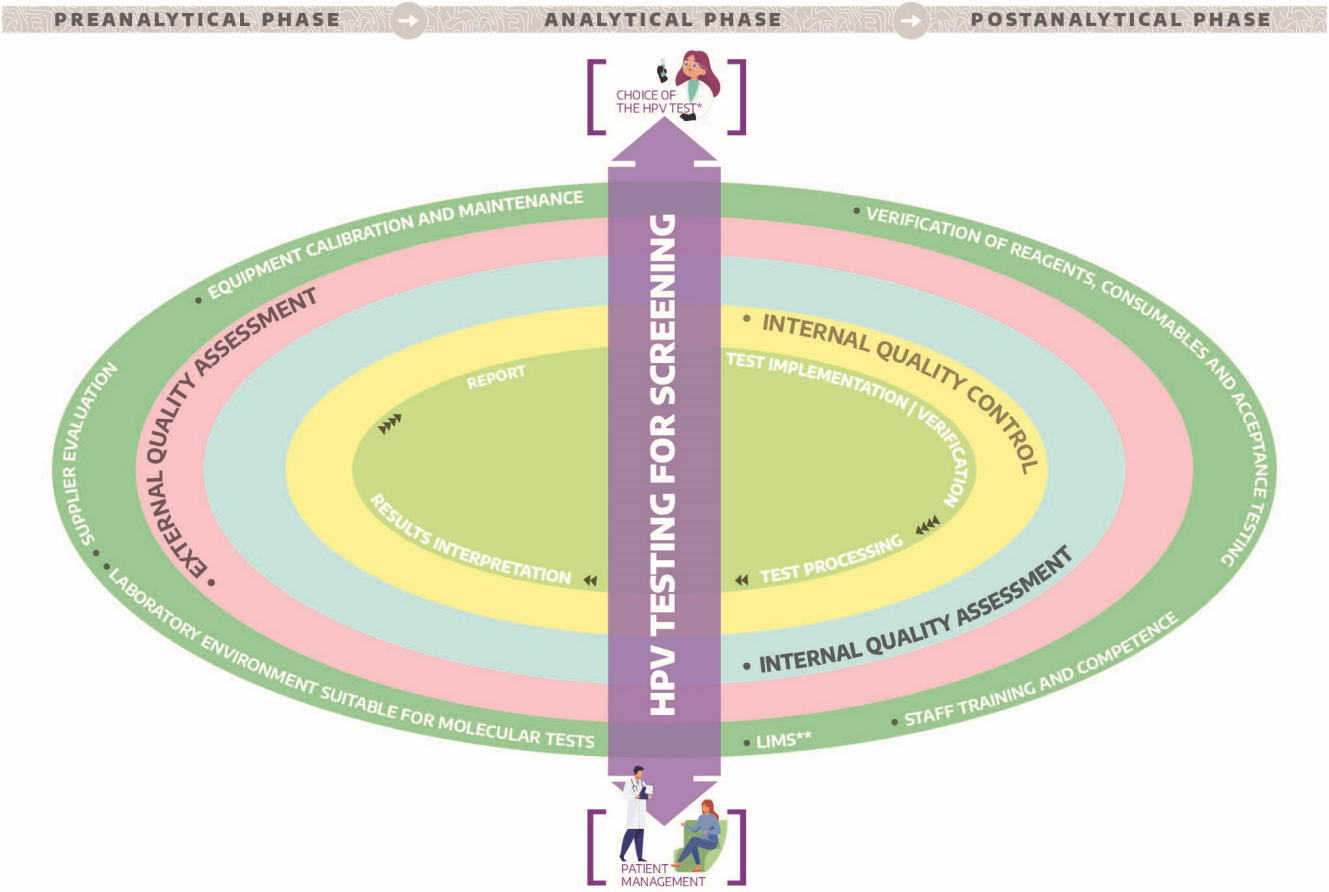
Quality assurance universe for human papillomavirus (HPV) testing in cervical screening. *Test analytically and clinically validated.*LIMS: Interfacing between HPV assay platform an the laboratory information management system.

In line with the remit of HPV LabNet to support the international community, particularly in view of the increased use of HPV testing for screening and disease management, this review summarizes the main points to consider when adopting quality assurance for HPV testing in primary cervical screening (Figure 1). In addition, strategies to facilitate its initiation and sustainability are also discussed. We hope this review will be of particular interest for settings and laboratories that are looking to include HPV in their scope of practice.

## IMPLEMENTING HPV SCREENING TEST

### Formats of Available Tests

HPV tests are based on the detection of viral nucleic acids in varied formats, including DNA-based and mRNA-based, with and without polymerase chain reaction (PCR) amplification. These target several different HPVs, including those most clinically relevant given their oncogenic potential. Of these, 12 types are classified as carcinogenic (group 1) by the International Agency for Research on Cancer (IARC) (HPV16, HPV18, HPV31, HPV33, HPV35, HPV39, HPV45, HPV51, HPV52, HPV56, HPV58, and HPV59); most tests also include HPV68 (group 2A ‘probably oncogenic’) and some HPV66 (group 2B ‘possibly oncogenic’).^11^ Around seven types (HPV16/18/31/33/45/52/58) confer a higher oncogenic risk than the others,^12^ and tests that can distinguish these seven HPV types are sometimes referred to as ‘extended’ HPV genotyping tests

The main differences between the screening tests relate to technology, including throughput level and availability of automation. Generally, screening tests can be divided into four groups^13^:

- HPV DNA tests that screen clinically the most oncogenic HPV types without distinguishing the individual genotype/s.
- HPV DNA tests with concurrent partial/extended, or reflex partial genotyping for the main genotypes.
- HPV DNA tests with full individual identification of the 12 oncogenic genotypes (or more).
- HPV mRNA tests that screen clinically the most oncogenic HPV types without distinguishing the individual genotype.

Speculation exists that HPV assays targeting the E6/E7 genes are more sensitive than those targeting the L1 gene because the latter may be disrupted in the viral integration process; however, there is little evidence from large-scale studies that L1 disruption affects sensitivity. In the meta-analysis by Arbyn et al, the relative sensitivity for CIN2/3+ compared with standard comparator HPV tests was very similar for either L1 or E6/7-targeted assays.^13^

### Clinical Validation

The key issue for HPV molecular testing in cervical screening is its ability to detect viral infections associated with cervical intra-epithelial neoplasia (CIN) grade 2 or higher (CIN2+), and to (ideally) delineate significant infections from transient infections that only require follow-up. Thus, unlike other molecular microbial tests, high-analytical sensitivity is not the aspiration, rather, it is the qualitative ascertainment of clinically relevant levels of HPV that makes a test fit for purpose in screening.

Meijer et al^14^ established international guidelines and minimum requirements for novel HPV tests regarding sensitivity, specificity, and reproducibility compared with the clinical performance of two tests that have been clinically validated in longitudinal screening trials: Hybrid Capture 2 (HC2; Qiagen, Gaithersburg, Maryland, USA) and GP5+/6+PCR enzyme immunoassay (GP5+/6+PCR EIA, Diassay, Rijswijk, The Netherlands).^14^ Notably, of the large number of HPV tests available (more than 200), relatively few have been validated following these criteria.^15^ At time of publication of this piece, the 2020 list of HPV assays that met Meijer 2009 criteria numbered around 10 which showed consistent equivalent accuracy (compared with the two standard comparator tests), and high intra- and inter-laboratory reproducibility as shown in multiple studies.^13^ Furthermore, a series of other assays have fulfilled some, but not all, the validation criteria and many more are under evaluation; therefore this will continue to be a dynamic list.

A complementary approach to assay validation is being undertaken by an international initiative, VALGENT (VALidation of HPV GENotyping Tests), created to support evaluation of HPV tests’ clinical performance, including those with genotyping capabilities.^16 17^ VALGENT uses stored samples for which longitudinal outcomes are known, which can expedite the process of validation.

While the Meijer 2009 criteria have been fundamentally important in setting a benchmark for suitable performance, future update and development will be welcome. The current guidance does not, for example, comment on suitable pre-analytical and operational aspects that would affect potential scalability. Laboratory professionals, clinicians, and public health decision-makers should be careful to choose assays associated with robust clinical validation and performance data. Kits approved by the US Food and Drug Administration and/or the European Medicines Agency (EMA) provide additional reassurance.

Likewise, it is important that the chosen HPV test(s) are clinically validated for their particular application such as cervical cancer screening and that they are used on appropriate biospecimens. To date, the most frequently used biospecimens for HPV testing are clinician-taken samples in cytology preservation media. All manufacturers of the commercial HPV tests that meet Meijer 2009 criteria include a claim for this type of sample within the instructions for use. A smaller number of tests include a formal claim for use on self-taken samples, although this is likely to change given the increasing demand for self-sampling.

### Assay Verification

The implementation of a new assay methodology has to undergo appropriate verification in each laboratory prior to routine use, even for commercially available HPV assays.^18^ Assay verification is the process of testing and reviewing an assay’s performance in relation to the assay manufacturers’ defined performance specifications, as stated in the package insert.^6^

The manufacturer is responsible for providing the analytical performance characteristics, as well as expected clinical performance data; this should reflect performance assessment relative to internationally accepted criteria for screening tests, as described earlier. Ongoing verification is also recommended, at least annually.^18^ It is important to note that if the user laboratory modifies the commercial assay and/or the instructions for the intended use, it is no longer considered an approved assay by the authorities, and the laboratory is expected to fully validate the impact of these changes on its performance.

### Endogenous Controls

Currently, most HPV assays contain an endogenous control; it frequently involves amplification of a housekeeping gene present in all human cells, such as β-globin, as a control of extraction and inhibition.^6^ While this can be helpful to confirm the presence of human cells, it does not confirm that the relevant cervical cells are necessarily present in the sample.^19^ On the positive side, these assays protect, to an extent, against the possibility of false negatives due to acellular samples. Even though this seems to be a rare event, it may arguably be more of an issue with the increasing use of self-sampling.^19^

### Individual Assay-run Interpretation

In each run, the clinical samples are processed along with the kit’s own controls and internal quality controls (see sectuion Internal Quality Control). After control checks, the results obtained for the clinical samples analyzed may be interpreted. Whatever the test, its results should be interpreted according to manufacturer’s instructions. Control results help to validate the run, and if the control result is not as expected, the run should be considered void.

In screening, HPV assays are generally applied at a qualitative level (presence or absence of HPV types at a manufacturer defined cut-off point) to make management decisions. The clinical usefulness and application of the semiquantitative read-out of the assay has not yet been demonstrated. As described earlier, detection of very small amounts of HPV using assays that have not been calibrated to disease endpoints is not helpful for screening purposes.

Laboratory operators should be aware of the ‘uncertainty of measurement’ of a test, which is the margin of doubt that exists about the result of any measurement, and also be aware of the critical factors that may affect this. The use of established, clinically validated tests supported by appropriately trained staff, and well-maintained equipment can mitigate uncertainty but not remove it completely. The core challenge/concern around HPV molecular screening tests (where there is an emphasis on clinical sensitivity and a negative result can trigger a recall of several years) will be uncertainty around the cut-off value resulting in false-negative results. However, as validated screening tests are tuned to achieve a high clinical sensitivity and are interpreted at a qualitative level, the level of acceptable ‘uncertainty’ for HPV tests may be greater than for other tests where a quantitative read-out would influence specific decision-making.

## QUALITY ASSURANCE: KEY CONCEPTS

Quality in healthcare is closely connected to the level of excellence in knowledge, and technical development. High quality molecular testing helps to ensure an accurate result is delivered to the right individual. The International Organization for Standardization (ISO)^20^ is an organization for standards development. An ISO standard is an internationally recognized way of performing a task. Having standards means that laboratories can work to the same set of guidelines no matter their location; this can support more consistent end results.

Accreditation is a good way to demonstrate the competence of a laboratory; to be accredited a laboratory undertakes a process whereby an authoritative body gives a formal recognition of technical competence for specific tests, based on third party assessment and following international standards. In diagnostics laboratories, the ISO 15189^21^ and ISO 17025^22^ are particularly relevant; the requirements of ISO 15189 cover the pre-analytical, analytical, and post-analytical phases, including standard operating procedures, the validation process, staff training, internal and external quality assurance, and laboratory set-up, among others.^23^ In some countries laboratory accreditation is (or will be) mandatory, but we should mention that this requirement does not include laboratories worldwide, including some in low- and middle-income countries.^24^ Notwithstanding formal accreditation, all laboratories should aim to implement a quality assurance framework. Quality assurance of test methods will be afforded by the combination of internal quality control and external quality assessment, among other aspects (Table 1).

**Table 1:** Key quality terms and processes

Term	Description	How does this help?	Comments
Laboratory Quality Management System (LQMS)	A framework of practices, procedures and policies that support the quality of laboratory test results.	Enables quality monitoring of the end to end test process. Identifies gaps and issues to allow appropriate alerts and mitigations.	For the overarching principles of LQMS, see Laboratory quality management system: handbook.^7^
Validation	A process and practice to ensure the test (and test system) is performing as expected *within a particular setting.*	Provides confidence, locally that the test/test system is robust in the hands of a specific team and environment.	Please note that the terms validation andverification are sometimes used interchangeably. However, the principles are sound: ensure the test is working as expected in a particular environment and then continue to monitor its performance. International performance criteria on which HPV test performance is adjudicated exist. If the test is to be used for routine cervical screening. It is recommended that a test which is clinically validated according to these criteria is used.^13^ Regulatory claims for tests labeled as ‘diagnostic’ can help provide confidence in the assay. However, local validation is important to ensure that the test is performing as anticipated in a particular laboratory environment. Application of diagnostic test that is used ‘off label’ in a way that deviates from the instructions for use requires validation.
Re-verification	A process to ensure the test continues to perform as anticipated *in perpetuity.*	Provides confidence that the test/test system is robust and working stably within a particular environment.	Re-verification schedule depends on local policy and real-time changes. Re-verification schedules may also depend on how frequently a test is performed and its complexity. Justification for the timing of a re-verification should be clear and reflect the demands and complexity of the specific test/process.
Internal quality control (samples)	A practice to ensure day to day consistency of a test in its ability to meet anticipated and acceptable performance.	Provides confidence that the test/test system is robust on a day to day basis, provides insight into real-time issues.	Independently sourced internal quality control samples should be run in addition to those that may be prescribed by the manufacturer according to instructions for use. Internal quality control samples can be produced locally or sourced from independent providers and should reflect the sample being assessed routinely as closely as possible. If the HPV assay provides a quantitative or semiquantitative output this can be plotted and assessed to monitor assay drift. Tolerances should be defined, and deviations investigated.
External quality assurance (external quality assessment) scheme	A scheme designed to confirm the accuracy of a test through provision of samples by an independent source, which is tested by the internal operators ‘blind’ to the expected result.	Independent assessment of assay performance. In addition to individual performance reports, the overall, international report generated by the scheme allows laboratories to assess and contextualize their performance relative to others.	For formal external quality assessment, the scheme provider controls the process of scheduling, dissemination, and scoring. For current examples of external quality assessment schemes see Table 2. Residual material from external quality assessment panels can support reagent-acceptance testing and re-verifications.
Inter-laboratory schemes	A scheme operated by a network of laboratories to determine test accuracy and consistency; can involve reciprocal exchange of material(s) or rely on provision of material from a central source/laboratory.	Can build relationships and dialog with partner laboratories. Can complement external quality assessment or serve as a proxy for external quality assessment if participation in a formal scheme is not possible. Can be made bespoke (in terms of biospecimen) for a particular. test/application	Inter-laboratory schemes may be particularly beneficial at the ‘start’ of a new service supported by one or more laboratory within a particular setting.

## INTERNAL QUALITY CONTROL

Internal quality control involves daily monitoring of the reproducibility or precision of the assay to detect errors in the analytical procedure.^25^ This is achieved using a control material of known content, which should yield results within predefined limits according to the validated technical characteristics of the assay, and supports the veracity of each run to ensure that the method works consistently day after day.^24^

### Types of Control Materials

Although commercial tests may include their own controls, internal quality control requires control materials, independent of those provided with the test kit, which can help evaluate variations between kit batches. The control materials should ideally resemble the biomatrix of samples, be prepared in sufficient quantity to last ideally at least 1 year, be stable for the period of use, be aliquoted for convenient use, and preferably be subjected to the whole procedure, from extraction to detection. Controls may be purchased from a commercial source, obtained from reference laboratories, or prepared in-house. Positive and negative HPV control materials can be generated in different ways, such as pooled from residual positive and negative HPV cervical samples, dilutions of HPV positive and negative cervical cancer cell lines in cytological media, and liquid, lyophilized, or freeze-dried specimens representing individual HPV types.^18 24 25^

### Application, Frequency, and Monitoring

Since HPV testing is applied at qualitative level, the internal quality control needs to involve both negative and positive HPV control materials, the latter optimally close to the test’s cut-off point. However, the possibility to register a numerical or semiquantitative measure, depending on the assay, permits the operator to analyze the trend in behavior of the assay. Therefore, the internal quality control is useful to check each run (as an additional control, independent of the own controls of each kit), kit batch, and process over time. It is advisable to include an internal quality control in each run/sample batch or at least every 24 hours or with a new kit batch, in cases of continuous platform instruments.^26^

Laboratories should monitor and log internal quality control performance, and acceptability criteria should be previously established. At the qualitative level, the match between expected and observed results must be analyzed. Additionally, in assays that provide a numerical read-out, recording values allows the creation of control charts (Levin-Jennings).^26^ Each laboratory may create its own control chart; there are also commercial providers that offer the ability to monitor the internal quality control.

### Analysis of Results and Corrective Actions

A control chart allows identification of a random error (deviation from an expected result) and provides information as to whether a particular run is acceptable. Additionally, systematic errors can drive a change in the mean value of the quality control material, either gradual or sudden, which may trigger the test procedure review, equipment calibration, and reagent performance. All laboratories should have their rejection rules and corrective actions documented.

## INTERNAL QUALITY ASSESSMENT

Internal quality assessment is the repeat testing of a percentage (typically 0.5–1% of the workload) of routine test samples to determine the laboratory’s ability to obtain reproducible results. Consistency is an important measure of quality assurance, repeat tests continually giving the same results as the original test provide evidence that a system is in control.^18 25 26^ This said, for samples where load is on assay cut off point and where there is no underlying disease, discrepancies may arise which may be challenging to manage. Creating pooled samples for internal quality assessment may mitigate this. Internal quality assessment is not mandated for ISO15189 provided a laboratory performs internal quality control and external quality assessment, although it may be a helpful option.

### Application, Frequency, and Monitoring

There are no specific recommendations for the number and/or frequency of internal quality assessment that should be implemen. For monitoring, the internal quality assessment result is compared with the original result and registered. Discrepancies may be noted and are normally classified as minor (a variation which would not affect the result/management) or major (lead to a different result/management). All discrepancies should be investigated. Factors that could affect the internal quality assessment result should be taken into account, like potential specimen degradation during storage and (if available) the semi-quantitiave value of the original result t.^24 27 28^

## EXTERNAL QUALITY ASSESSMENT

External quality assessment, sometimes used interchangeably with proficiency testing, is defined as a system that allows comparison of a laboratory’s testing performance with a source outside the laboratory.

### Interlaboratory and Interlaboratory Exchange Schemes

Interlaboratory schemes involve reciprocal exchange of material of known quantity or may involve the creation of a pool of often ‘blind’ material(s) by a central laboratory that are then disseminated to participating laboratories. In both scenarios, observed versus expected results are assessed, with discrepancies noted and investigated. Interlaboratory exchange schemes can complement formal external quality assessment schemes and can help to build robust relationships and communications between laboratories, particularly within a particular country/service. They may also be particularly valuable at the initiation of a new service—for example, in England, prior to the national introduction of HPV testing for triage and test of cure, the relevant service laboratories were sent a panel of centrally collated and annotated samples suitable for testing on the varied HPV platforms approved for use within the program.^18^ This said, in the authors’ experience, the level of formality of interlaboratory exchange schemes varies from being resourced, mandated and monitored by program ‘oversee’ to a more grass-roots approach by cooperating laboratories.

Additionally, interlaboratory exchange schemes can add value if existing external quality assessment schemes do not reflect the biomatrix of the sample routinely used for testing. If a laboratory is looking for accreditation by an external agency, then interlaboratory exchange schemes is not generally mandated provided participation in a recognized external quality assessment scheme is demonstrated.

### External Quality Assurance

External quality assessment schemes have commonalities with interlaboratory schemes, although they operate independently of a setting, are often (not always) associated with external accreditation, and are disseminated, as panels, through defined and specific cycles. Although many are not-for-profit, a charge is usually levied, and sometimes international distribution can be restricted; this can be a reason for pursuing an interlaboratory exchange schemes approach. Table 2 shows examples and key details of currently available schemes for HPV screening, although we accept that this table may not be entirely comprehensive Notwithstanding this, existing external quality assessment schemes vary in size and dimensions, frequency of distribution, and scoring system. Clearly participation in more than one scheme is possible and is advised if a laboratory has a diverse remit that includes provision of genotyping information as well as qualitative results (i.e. presence or absence of high risk types). In recent years, available external quality assessment schemes have diversified with respect to the biomatrixes available, which is welcome. Panels now reflect specimens in viral transport media, cytology-preservative media, and formalin-fixed paraffin-embedded material. The majority of schemes report the qualitative presence or absence of high-risk HPV, which is justifiable given that this is the result that generally triggers management.

**Table 2:** Overview of human papillomavirus independent quality schemes

Name of external quality assessment provider	General dimensions (size and distribution)	Biosample type	Scoring	Web link	Comments (include accreditation status)
Quality Control in Molecular Diagnostics	Panel of 12 samples 1 x year Or 2 Panels 6 samples 2 x year.	ThinPrep and Surepath.	Qualitative presence/absence of high-risk HPV types. Performance adjudicated at ‘analytical’ and/or ‘clinical’ level.	http://www.qcmd.org/ 31	Distribution may contain educational samples. Accredited to ISO 17043
College of American Pathologists Quality Solutions	Varied schemes offered (see web link) but for liquid media generally 5 samples 3 x year.	ThinPrep, Surepath, and ‘mixed media’ and Digene transport medium. For in situ hybridization external quality assessment: slides.	Scheme dependent: includes qualitative detection of high-risk types and type-specific assessment.	2023-CAP-Surveys-Catalog 32	Participation in some of the schemes may be limited to USA. Accredited to ISO 17043
International HPV Reference Center	One typing panel/year (44 samples). One screening panel/year (12 samples).	100 μL of TrisEDTA buffer containing human placental DNA (10 ng/μL). Users are to dilute them with 1 mL of their corresponding media for testing (eg, ThinPrep).	Qualitative presence of HPV types. No false positivity allowed.	https://www.hpvcenter.se/proficiency_panel/ 33	Proficiency typing panel contains 3 samples comprising cell suspensions to allow evaluation of DNA extraction methods. International units are used (HPV16,18)
Quality In Pathology - ‘QuIP’	One panel	Formalin-fixed, paraffin-embedded sections simulating head and neck lesions.	Type specific presence of high risk and low risk HPV.	Quality in Pathology – QuIP: Proficiency Tests 34	Submitted for accreditation to ISO 17043
UK National External Quality Assurance Service (UK Nexternal quality assessmentS)	4 samples 3 x year	PreservCyt	Qualitative presence/absence of HR types Type specific details provided for information rather than scoring.	https://ukneqasmicro.org.uk 35	Accredited to ISO 17043
LABQUALITY	2 samples, 4 x year	‘Simulated’ cervical samples.	Qualitative presence/absence of HR types.	Human papillomavirus, nucleic acid detection %7C Labquality 36	Accredited to 17 043 (PT02/FINAS) Distributions to Europe, Middle East, and Asia

We have restricted this table to schemes that are available to external users and accessible by those outside the country of collation/origin. Operation of internal schemes within a particular region and/or country is of course feasible and often attractive, particularly at the start of an HPV service: examples of this are in China,^37^ Norway,^38^ and Australia.^39^

External quality assessment schemes should ideally be amenable to a variety of HPV tests to reflect the international diversity and availability of HPV tests, with their respective chemistries and targets; any doubt may be discussed with the scheme coordinator. Ideally, the external quality assessment schemes used should challenge the end-to-end process of a test, including any pre-analytics and extraction as well as detection.

### Analysis of Results and Corrective Actions

The results of external quality assessment should be clearly documented and made available internally and to users of the service, where required. A (minimum) yearly review of external quality assessment performance is advised, although any non-conformance should be managed in real time with a documented root cause investigation and suggested corrective and preventative actions. It is important during the investigation (of discrepant results) to reflect on the *overall* report provided by the scheme provider as well as the individual laboratory report, as this can help contextualize individual laboratory performance with that of the wider community experience and may help to highlight issues with a particular platform. Recourse to the scheme provider can be helpful during the investigation process for gaining advice and, potentially, for provision of additional residual material should retesting be of value for troubleshooting purposes

## Annual Quality Assurance Data Analysis

Annual review of quality assurance is intended to provide an update and overview of the whole process of the HPV molecular test repertoire,. It should include the profound assessment of the following topics:

### Quality Control

Results of the internal quality controls as well as of external quality assessment should be assessed with a focus on exceptions that may indicate quality problems. Comparison of results with those of previous years, together with a check on existing performance trends, should be in place.

### Supplier Assessment

A review of supplier activities should include an assessment of (a) suppliers of laboratory materials (devices, reagents, control materials, small consumables), (b) suppliers (organizers) of external quality assessment schemes, and (c) suppliers of analytical results when subcontractors are used.

### Assessment by Auditors

Relevant issues/findings from internal and external audits should be described. including installing corrective actions

### Assessment of Feedback

Reflection on the user’s perception of the services provided by the laboratory should be performed. The laboratory should also formulate an action plan informed by user feedback.

### Non Conformities

Deviations from procedures (or non-conformities) must be documented throughout the year, and these records are the basis for the annual overview, including the observed trends and evolutions within previous years as well as the corrective actions applied.

### Technical Evolution and Staff Training

Improvement proposals by the technical staff as well as evaluations of current technical needs, including improvement of laboratory infrastructure, should be performed. The training needs of technical staff, including effectiveness of existing training plans, should be evaluated.

### Quality Indicators

Quality indicators should be monitored to evaluate critical aspects of the pre-analytical, analytical and post-analytical phases. If feasible, parameters should be quantifiable to evaluate the nature and magnitude of corrective actions applied, eg. number of corrected reports. The most used measurable quality indicator is turnaround time (TAT) i.e. the *time interval from sample reception to the availability of the test result*. The TAT should be established in accordance with practical capacities of the individual laboratory as well as actual clinical needs and will strongly depend on the global positioning of HPV testing in the particular country/laboratory (primary cervical cancer screening, cytology triage, therapeutic follow-up etc.).

### Risk Management

The impact of the work processes, and if applicable the implications of their disruption/ failure on patient safety should be evaluated. Identified risks should be reduced or eliminated and all decisions about the corrective actions should be documented.

## ADDITIONAL ASPECTS OF LABORATORY PRACTICE

### Molecular Testing and Laboratory Environment

We accept that access to a bespoke laboratory space which has been configured with molecular testing in mind may be a luxury. Additionally, how rapidly a new platform can be integrated will depend on various factors. including the extent of any overarching laboratory quality management system. In Table 1 we summarize and reflect on some of the key processes that can support quality testing.

Most HPV tests in use for screening and clinical management are based on molecular amplification. The enemy of molecular testing is contamination and having systems and protocols in place to manage this is important; some HPV assays integrate digestion of amplified products, but this alone is not a solution. Should the assay require, in-house preparation of primers and master-mixes restricted ‘clean’ preparation areas that contain dedicated laboratory coats, small equipment (vortex, microfuge, pipettes, reagent freezers), and a local standard operating procedure for appropriate use are needed. Records that detail preparation of primer and master-mix batches should be completed to ensure traceability and support troubleshooting in the event of downstream problems. The higher throughput, clinically validated, HPV assays are increasingly less reliant on manual preparation steps. Additionally, detection of PCR products in real time obviates the need for exposing ‘open’ PCR products to the environment.

To summarize, the extent of molecular demarcation will be affected by the nature of the assay used. However, even with automated analyzers, introduction of a cleaning and decontamination schedule(s) appropriate to a chosen test (cleaning products that can destroy nucleic acid safely but will not interfere with the specific reaction chemistry) will support good laboratory practice, as will the creation of a policy and practice for waste management. Standard operating procedures should include such aspects on decontamination.

### Assessment of Reagents and Acceptance Testing

Even with well-established, commercial, clinically validated HPV assays, it is important that the impact of changing reagent-lots on test performance is monitored and that particular lots are confirmed as fit for purpose through acceptance testing. A gradual trend in the growth of use of commercial tests for HPV in European Union countries by 2028 is expected.^29^ While there is no international standard for acceptance (criteria should be decided locally) the addition of extra quality controls or previously tested, pooled samples can help to provide confidence in a lot. Records of acceptance should be available with clear sight of the operator, the lot number, confirmation of the version of instructions for use/kit insert, summary of the results, and date the lot is put into active use. Users should not forget to include all reagents used for testing, including those not included in the commercial kits. Monitoring lot performance can help to inform discussions with manufacturers in cases of unexpected performance.

### Equipment: Calibration, Maintenance, and Interaction with Manufacturer

Small and large equipment used to deliver a particular test, whether provided by the test manufacturer, or required but not supplied (such as pipettes and cold storage), need to be well maintained. Maintenance schedules can be created to support timely organization of preventative maintenance by external or internal personnel. Documentation of maintenance and repair should be detailed and include dates, activities, and personnel involved. A daily, operational user log associated with a particular piece of equipment can support team communication about problems, particularly if the equipment is multi-user. Additionally, environmental records of ambient temperature and the temperature of any associated equipment used for storing reagents or controls relevant to the equipment/test should be documented daily, with lower and upper tolerances specified, exceptions recorded, and associated corrective actions documented. A system/method to ensure that the temperatures are accurate within the required range through external calibration is also required. Additionally, prior to receipt of reagents by a laboratory, the appropriate transport of commercial reagents should be secured by the manufacturer.

A check to see that the assay is performing as expected should occur after preventative maintenance. The process used to provide evidence for this—sometimes referred to as a ‘back to service’ exercise—should be documented and available for view. Additionally, for significant ‘changes’ such as a physical movement of the equipment within the laboratory, after a critical repair, or after a software update, reverification of the assay is advised.

### Supplier Evaluation

Good relationships and communications between the commercial supplier of the HPV platform and the operating laboratory are important; initial training on a particular assay should ideally be supported by the supplier, and documentation of this training made available. The obligations for service and maintenance of any test equipment should be honored according to the maintenance contract. As described earlier, an annual documented review of any problems with suppliers (including response times) is important to identify particularly areas of pressure and challenge and to inform subsequent improvements.

### Interfacing Between HPV Assay Platforms and Laboratory Information Management System

Frequently, in cases of high-throughput analyzers, automatic interface of the machine output with the laboratory information management system occurs, which supports rapid generation of results and can obviate transcription errors associated with manual input. However, if an interface is created, clear demonstration of its accuracy should be trialed and documented before it is put into live practice.

## STAFF TRAINING AND COMPETENCE

Competence is the demonstrated ability to apply knowledge and skills that allow staff to perform in specific work situations in accordance with the occupational area standards.^7^ In a quality assurance program, having the staff trained and continuously updated is a central component. Staff should know the objective of the test being performed, its technical basis, the critical processing points, criteria for the use of reagents (at the time of testing and storage) and equipment (use and maintenance conditions), and sample handling, with special care to prevent contamination.

Staff should have clearly defined lines of responsibility and job descriptions. Likewise, it is important to ensure that the personnel are aware of the relevance of their activities, and their contribution to meeting quality objectives.^24^ The responsible authorities must guarantee the competence of the personnel and the effectiveness of any training provided to maintain it, so that the quality of the result is not affected. If HPV testing is performed in a screening area that is not part of a virological/clinical laboratory, it is recommended that a professional, with proven laboratory competence and experience in the field has a supervisory role (according to ISO15189).^21^

If a laboratory decides to adopt more than one HPV testing method, the personnel must be trained for each of the tests used.^27^ In addition, the general principles of good laboratory practice that underpin quality testing must be taken into account.^30^ There must be evidence that training is provided on site and that the personnel performing the tests have completed their training and will be supported in maintaining their competence, again, clear and documentation of this evidence is key.^27^

## CONCLUSIONS

The aim of this work was to provide laboratories with an overview of core quality control measures that can help to ensure robust laboratory-based HPV testing. Quality monitoring of HPV testing is an evolving field, and developments in (1) the tests themselves, (2) the associated automation, (3) international validation metrics, and (4) regulatory requirements will probably influence and inform the approach to quality monitoring over time. We also accept that globally, laboratory infrastructure and remit varies widely and that the implementation of some of the items described above may be challenging. Nevertheless, we hope that this piece introduces some of the key concepts on quality assurance of HPV testing and its importance. Given the high negative predictive value of HPV testing which lends itself to extended screening intervals of 5 years, accuracy, supported by quality, is clearly fundamental. We would also argue that quality assurance should be a prime consideration of any laboratory looking to undertake HPV testing and that creation and maintenance of a core local policy to support this must be implemented according to each environment.

Certainly, the requirement for increased HPV testing globally is indisputable; as one of the three pillars of cervical cancer elimination described by WHO, it is estimated that around 1.5 billion tests over a period of 5 years will be required to reach the 70% ‘target’ of screening women twice in their life time. Ensuring these tests are delivered accurately to the right person at the right time is not a trivial undertaking and quality frameworks to support this will continue to be essential.
